# Impact of multimorbidity on healthcare costs and utilisation: a systematic review of the UK literature

**DOI:** 10.3399/bjgp20X713897

**Published:** 2020-12-01

**Authors:** Marina Soley-Bori, Mark Ashworth, Alessandra Bisquera, Hiten Dodhia, Rebecca Lynch, Yanzhong Wang, Julia Fox-Rushby

**Affiliations:** King’s College London, School of Population Health Sciences, London.; King’s College London, School of Population Health Sciences, London.; King’s College London, School of Population Health Sciences, London.; King’s College London, School of Population Health Sciences, London.; King’s College London, School of Population Health Sciences, London.; King’s College London, School of Population Health Sciences, London.; King’s College London, School of Population Health Sciences, London.

**Keywords:** depression, healthcare costs, healthcare use, multimorbidity, primary care, systematic review

## Abstract

**Background:**

Managing multimorbidity is complex for both patients and healthcare systems. Patients with multimorbidity often use a variety of primary and secondary care services. Country-specific research exploring the healthcare utilisation and cost consequences of multimorbidity may inform future interventions and payment schemes in the UK.

**Aim:**

To assess the relationship between multimorbidity, healthcare costs, and healthcare utilisation; and to determine how this relationship varies by disease combinations and healthcare components.

**Design and setting:**

A systematic review.

**Method:**

This systematic review followed the bidirectional citation searching to completion method. MEDLINE and grey literature were searched for UK studies since 2004. An iterative review of references and citations was completed. Authors from all articles selected were contacted and asked to check for completeness of UK evidence. The National Institutes of Health National Heart, Lung, and Blood Institute quality assessment tool was used to assess risk of bias. Data were extracted, findings synthesised, and study heterogeneity assessed; meta-analysis was conducted when possible.

**Results:**

Seventeen studies were identified: seven predicting healthcare costs and 10 healthcare utilisation. Multimorbidity was found to be associated with increased total costs, hospital costs, care transition costs, primary care use, dental care use, emergency department use, and hospitalisations. Several studies demonstrated the high cost of depression and of hospitalisation associated with multimorbidity.

**Conclusion:**

In the UK, multimorbidity increases healthcare utilisation and costs of primary, secondary, and dental care. Future research is needed to examine whether integrated care schemes offer efficiencies in healthcare provision for multimorbidity.

## INTRODUCTION

With improvements in public health and access to good-quality care, people are living longer but frequently with multimorbidity. Multimorbidity, often defined as the coexistence of two or more conditions,^1^ challenges quality improvement and cost-containment efforts. In 2015, 54% of people aged >65 in England exhibited multimorbidity; this percentage is projected to increase to 68% by 2035.^2^ The current single disease-oriented model of care delivery struggles to address the needs of patients with multimorbidity, who often experience care fragmentation, difficulty in managing their treatments, and poor health outcomes.^3^^–^^6^ The Quality and Outcomes Framework (QOF), a quality improvement programme available to all GP practices in England since 2004, links payments to 77 indicators reflecting public health and clinical targets.^7^ However, as it takes no account of multimorbidity,^8^^–^^10^ GPs are not incentivised through this significant mechanism to focus on multimorbidity.

Besides quality of care shortfalls, multimorbidity may also result in higher healthcare utilisation and costs compared with single health conditions.^11^ Lehnert and colleagues^11^ systematically reviewed 35 studies that investigated the relationship between multimorbidity and healthcare costs and utilisation. They showed that costs and utilisation (including physician visits, hospitalisations, and medication use) tend to increase with the number of conditions. Lehnert *et al* ’s review,^11^ conducted in 2010, did not find any UK studies on this topic. The relationship between multimorbidity and healthcare costs and utilisation, particularly its magnitude, may vary not only by person-specific and environmental factors (such as frailty, income deprivation, or availability of social care services), but also across health systems.^12^^,^^13^

The aim of this review was to describe the relationship between multimorbidity and healthcare costs and utilisation in the UK; and to identify whether this relationship varies by disease combinations and healthcare components.

## METHOD

This systematic review followed the bidirectional citation searching to completion (BCSC) method. BCSC starts by selecting an initial set of relevant studies (‘pearls’), based on expert knowledge or a systematic literature review, followed by a review of references and citations of the ‘pearls’ to gather further appropriate literature. After excluding irrelevant studies from the reference and citation search, this process is repeated until no further sources are found. BCSC mirrors snowballing of citation searches forward and backward, and iteratively repeats this process until no further studies are identified. Although rarely used as the primary method of systematic searching, BCSC may be an equally effective technique to comprehensively gather studies as a conventional systematic literature review.^14^^,^^15^

**Table:** How this fits in

Multimorbidity, the presence of two or more conditions, is becoming the norm rather than the exception in primary care. This review of 17 UK studies has drawn attention to both the high service utilisation and cost of providing health care to patients with multimorbidity, particularly when depression is one of the conditions. One unanswered question is whether models of ‘integrated care’ might mitigate the high cost of care.

To identify the initial list of pearls, the authors’ initial knowledge of studies was supplemented by a Boolean logic search on MEDLINE (see Supplementary Appendix S1 for details). The query combined terms used in the National Institute for Health and Care Excellence (NICE) multimorbidity guidelines,^5^ MEDLINE UK filter,^16^ and two systematic literature reviews on multimorbidity.^1^^,^^17^ The NICE Evidence Search catalogue,^18^ Scottish Intercollegiate Guidelines Network,^19^ and the website of the International Research Community on Multimorbidity^20^ were also used to identify additional publications and grey literature.

Two authors independently reviewed the first 100 titles and abstracts. The study inclusion and exclusion criteria (Box 1) were further refined after discussing discrepancies, and a second double review of 100 sources was conducted. The first author screened the remaining articles. To target original research testing the relationship between multimorbidity and healthcare costs and utilisation, descriptive cost-of-illness, economic burden, or cost-effectiveness studies were excluded, along with literature reviews, meta-analyses, and study protocols. Results of the search and selection are reported in accordance with PRISMA guidelines.^21^ The final list of selected articles was shared with the corresponding author of each article to check for comprehensiveness.

**Box 1.** Inclusion and exclusion criteria

**Inclusion criteria**	**Exclusion criteria**
Original research UK study Focused on assessing the relationship between multimorbidity and healthcare costs/utilisation as stated in the title or the study goal in the abstract Published after 2004^a^	Non-human research Descriptive cost-of-illness or economic burden studies, literature reviews, or meta-analyses (unless meets inclusion criteria),^b^ cost-effectiveness studies, or study protocols Study population is limited to a single condition, or a single condition with a procedure, risk factor, or complication of the single condition

a The 2004 threshold corresponds to the year when the Quality and Outcomes Framework was implemented and the NHS began the deployment of improved computerised applications for clinical records and diagnoses.

b In this case, references were searched for additional primary studies.

Data extraction and analysis focused on the study aims, definition of multimorbidity, justification of analytic framework, and econometric techniques to estimate cost and utilisation models, findings, stated limitations, and research gaps. Risk of bias was assessed using the National Institutes of Health’s National Heart, Lung, and Blood Institute quality assessment tools for observational cohort and cross-sectional studies.^22^ After piloting the data extraction form, two authors extracted data on a randomly selected 10% of studies to check for consistency, and the first author extracted the remainder. The results were grouped by healthcare cost or utilisation study type, tabulated (see Supplementary Table S1 for details), and reported narratively. Multimorbidity parameter estimates, which quantify multimorbidity’s relationship with costs and utilisation, were gathered and systematically presented for analysis. The heterogeneity among studies was assessed using *I*^2^, and data were pooled in a meta-analysis when possible.

## RESULTS

**Figure 1.**
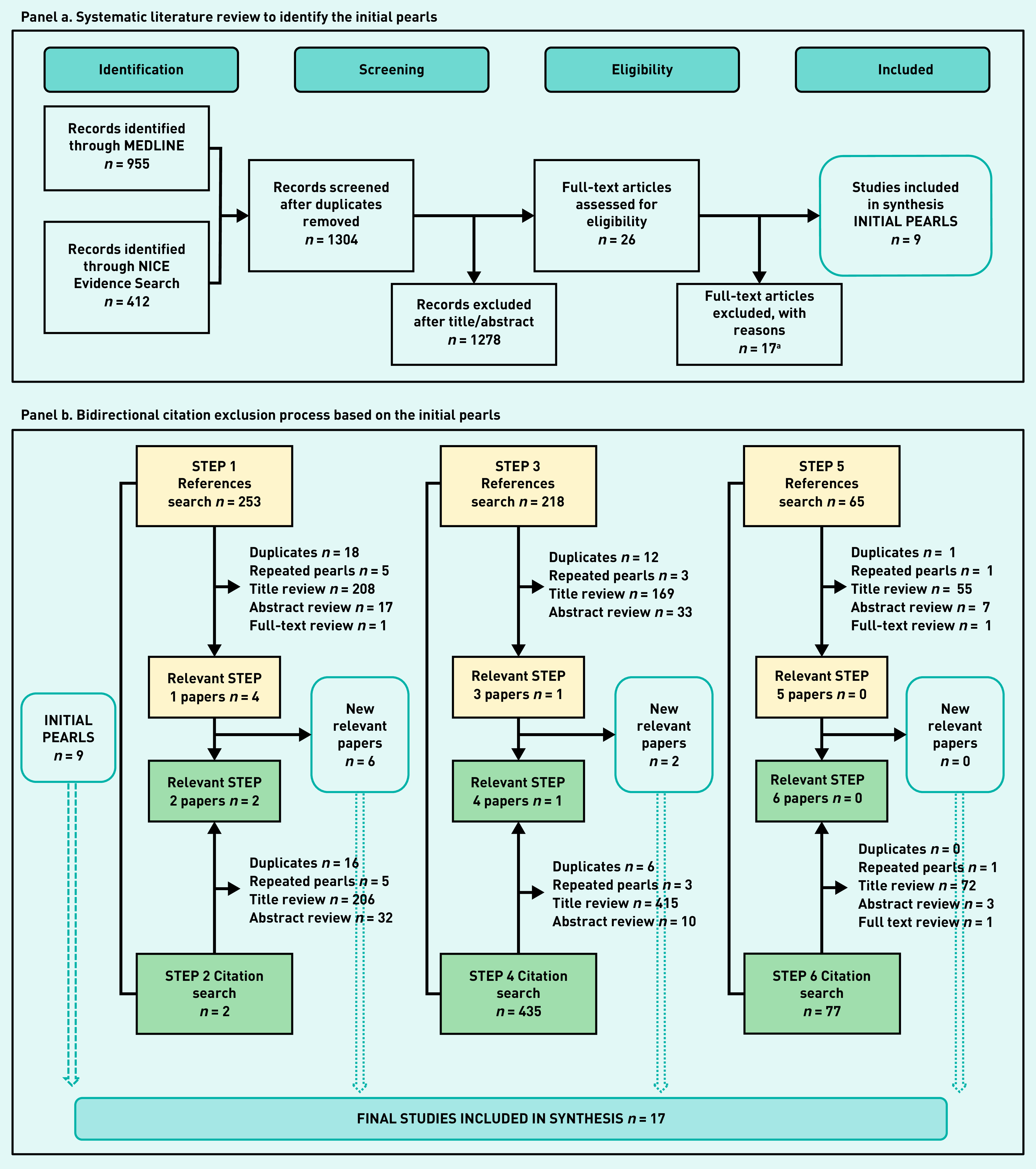
**Flowchart illustrating the search process.** ^a^**See Supplementary Appendix S2 for the list of these 17 excluded full-text articles with reasons.**

The review identified 1304 articles from the electronic searches, excluding duplicates. A total of nine articles (initial ‘pearls’) met the inclusion criteria after title, abstract, and full-text review (Figure 1, Panel a). By inspecting the references and citations of the initial pearls, eight more studies were selected (Figure 1, Panel b), producing 17 studies for synthesis (see Supplementary Appendix S3 for details).^3^^,^^13^^,^^23^^–^^37^ Contact with study authors (65% response, *n* = 11) produced no further studies.

### Study aims and data

The relationship between multimorbidity and healthcare utilisation was explored in 10 studies, while seven studies tackled multimorbidity and costs. Six studies covered the UK, nine focused on England, and two on Scotland. Both cross-sectional and longitudinal study designs were used, with up to 8 years of participant follow-up. The average sample size was 210 495 individuals (range 419 to 819 590)^23^^,^^24^ among the utilisation studies and 109 746 individuals (range 39 381 to 282 887)^25^^,^^26^ for the cost studies.

### Definition of multimorbidity

**Figure 2.**
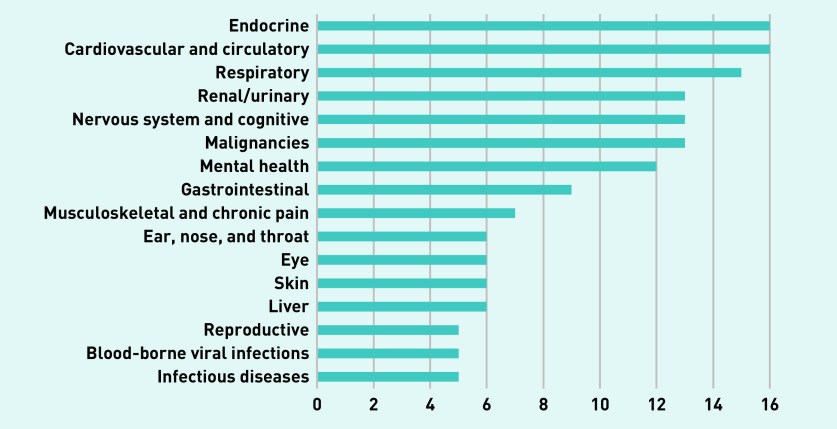
**Number of studies that included each body system in their multimorbidity measures.**^a^ ^a^**Medical conditions were grouped into body systems to facilitate data display (see Supplementary Appendix S4 for more details). This graph excludes one study,^37^ which did not detail the 49 conditions included in its multimorbidity measure.**

Large variability in the type of diseases considered to create the multimorbidity or condition count indicators was observed (see Figure 2 for details). All studies included conditions pertaining to the endocrine, and cardiovascular and circulatory systems. However, only a few (*n* = 5) considered the reproductive system or infectious diseases. QOF conditions were used in five studies. The number of diseases included in the multimorbidity or disease count measures ranged from four to any (see Supplementary Appendix S4 for details). For example, Charlton *et al*^26^ only considered coronary heart disease, stroke, colorectal cancer, and diabetes, while Payne *et al*^27^ included 40 conditions covering almost all body systems. Most studies did not provide an explicit definition of multimorbidity; six studies formally defined multimorbidity as two or more conditions. Two studies only considered long-term conditions to build their multimorbidity measures, while six studies focused on chronic conditions.

### Characteristics of the studies on multimorbidity and healthcare utilisation

Some focal points of multimorbidity and healthcare utilisation studies included the interplay among multimorbidity, deprivation, and utilisation, the combination of mental and physical conditions, the effect of multimorbidity among individuals with a long-term condition, and the comparison of alternative multimorbidity measures (see Supplementary Table S1, panel a for details).

Most studies (six out of 10) explored the determinants of unscheduled care use, including emergency department visits and hospital visits. Three studies aimed to explain primary care utilisation, while one study explored dental care use. Seven studies applied a retrospective cohort study design, while cross-sectional (*n* = 2) and prospective cohort study designs (*n* = 1) were used in the remaining studies. Six studies presented a justification for their analytic framework, including a study hypothesis (*n* = 6) or a reasoning behind the utilisation model specification (*n* = 3). Most utilisation models were calibrated using binary (use/non-use) logistic regression (*n* = 7). Other multivariate regression techniques included ordinary least squares (OLS) with a log-transformed dependent variable, generalised linear model (GLM) with a log-link and a negative binomial distribution, and a negative binomial regression. The most common predictors were age, sex, and deprivation. Other less common independent variables were education level, smoking status, distance to nearest hospital, and patient satisfaction. Three studies assessed the goodness of fit of the utilisation models.^28^^–^^30^

Multimorbidity contributes to higher healthcare utilisation, except for prolonged hospital stay among the oldest patient group (≥90 years) (Table 1). Patients with four or more conditions have almost 15 times the odds of experiencing an unplanned potentially preventable hospitalisation (odds ratio [OR] = 14.38) (Table 1).^27^ The combination of mental and physical conditions particularly increases the probability of unplanned hospital care to between 58% and 100%.^27^^,^^30^^,^^35^ In primary care, having multimorbidity, defined as two or more morbidities, more than doubles its expected use (OR = 2.56) compared with having 0–1 morbidities (Table 1).^3^ Adding a multimorbidity measure to a primary care utilisation model already accounting for age, sex, deprivation, and GP practice fixed effects notably improves goodness of fit (*R*^2^ increased from 0.22 to 0.37 with adjusted clinical groups (ACG) categories or to 0.42 with number of prescribed drugs).^29^

**Table 1.** Summary of the relationship between multimorbidity, costs, and utilisation^a^

**Utilisation/cost type**	**MM specification**		**Magnitude (95% CI)**	**Parameter estimate type**	**Reference**
**Primary care visits** (*n* = 3)					
	Number of QOF LTCs	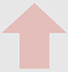	**0.37** (0.36 to 0.38)	Marginal effect	28
	MM vs not	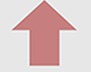	**2.56** (2.48 to 2.64)	Odds ratio	3

**Dental visits** (*n* = 1)					
	MM vs not	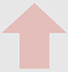	**1.23** (1.08 to 1.38)	Odds ratio	36

**A&E visits** (*n* = 2)					
	HADS score of 8 or more vs lower	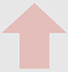	**1.58** (1.04 to 2.41)	Odds ratio	35
	1 QOF LTC vs none	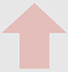	**1.12** (1.10 to 1.13)	Odds ratio	24
	2 QOF LTC vs none	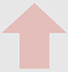	**1.28** (1.25 to 1.31)	Odds ratio	24
	3 QOF LTC vs none	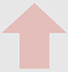	**1.65** (1.59 to 1.71)	Odds ratio	24
	≥4 QOF LTC vs none	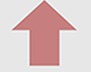	**2.55** (2.44 to 2.66)	Odds ratio	24

**Hospitalisations** (*n* = 4)					
All	1 LTC vs none	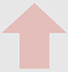	**1.77** (1.59 to 1.98)	Odds ratio	33
	2 LTC vs none	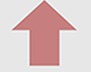	**2.41** (2.12 to 2.72)	Odds ratio	33
	3 LTC vs none	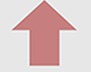	**3.53** (3.06 to 4.07)	Odds ratio	33
	≥4 QOF LTC vs none	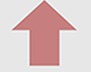	**4.33** (3.63 to 5.17)	Odds ratio	33
	MM vs not	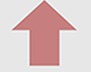	**2.58** (2.48 to 2.69)	Yearly rate ratio	3

Unplanned all	1 PC vs none	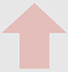	**1.70** (1.59 to 1.82)	Odds ratio	27
	2 PC vs none	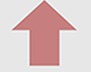	**2.69** (2.50 to 2.89)	Odds ratio	27
	3 PC vs none	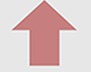	**3.47** (3.21 to 3.76)	Odds ratio	27
	≥4 PC vs none	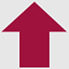	**5.87** (5.45 to 6.32)	Odds ratio	27

Unplanned potentially preventable	1 PC vs none	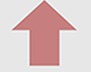	**2.50** (2.07 to 3.03)	Odds ratio	27
	2 PC vs none	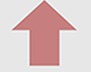	**4.93** (4.06 to 5.99)	Odds ratio	27
	3 PC vs none	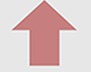	**6.82** (5.55 to 8.37)	Odds ratio	27
	≥4 PC vs none	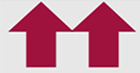	**14.38** (11.87 to 17.43)	Odds ratio	27

Prolonged length of stay	MM vs not (90+ population)	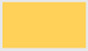	**0.61** (0.32 to 1.13)	Risk ratio	23

**Total costs** (*n* = 3)					
	1–3 LTC vs none	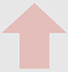	**1.62** (1.28 to 2.03)	Mean ratio	31
	4–6 LTC vs none	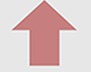	**2.53** (2.01 to 3.19)	Mean ratio	31
	7–9 LTC vs none	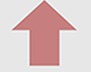	**3.82** (3.01 to 4.85)	Mean ratio	31
	1 LTC vs none	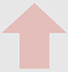	**1.99** (1.95 to 2.03)	Mean ratio	26
	2 LTC vs none	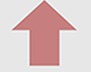	**2.53** (2.46 to 2.58)	Mean ratio	26
	3 LTC vs none	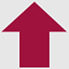	**2.86** (2.72 to 3.03)	Mean ratio	26

**Care transition costs** (*n* = 1)	Comorbidity pairs vs index LTC	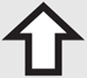	*P*<0.001	Increasing trend in association	34

**Primary care costs** (*n* = 2)	Costs of 1 patient with 2	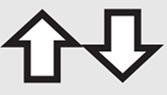	Increasing or decreasing costs when co-occurring	Estimated prevalence- adjusted cost	13
	LTC vs 2 separate				
	patients with each LTC				

**Hospital costs** (*n* = 1)	Individual LTC	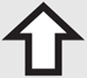	*P*<0.01 for 90% of the estimated coefficients	Estimated coefficient	25
	Time to death as a proxy for morbidity				

a The number of articles is indicated in parentheses next to the cost or utilisation type (see Supplementary Appendix S3 for the complete 17 study references). Mean ratios can be obtained by exponentiating the parameter estimates from a generalised linear model with the log-link; they have an interpretation similar to an odds ratio. For example, individuals with 7–9 conditions have 3.82 times the mean expected total costs of individuals without comorbidities. A&E = accident and emergency. CI = confidence interval. HADS = Hospital Anxiety and Depression Scale. LTC = long-term condition. MM = multimorbidity. PC = physical condition. QOF = Quality and Outcomes Framework. Prolonged length of stay is defined as 7 days in the hospital. Care transitions are defined as healthcare changes from general practice to emergency department or hospital care.

Review Manager (version 5) was used to calculate the overall effect of multimorbidity on healthcare utilisation. Results from the random effects model (see Supplementary Appendix S6 for details) suggest that people with multimorbidity are expected to use health services 2.56 times more than people without multimorbidity (OR = 2.56; 95% confidence intervals = 1.88 to 3.47). An *I*^2^ of 99% indicates considerable heterogeneity among the studies, which highlights that the meta-analysis results should therefore be considered with caution.

### Characteristics of the studies on multimorbidity and healthcare costs

Exploration of multimorbidity and healthcare costs included the interplay between multimorbidity and deprivation, the cost impact of specific disease combinations, the relationship between age, time to death, and multimorbidity, and the comparison of alternative multimorbidity measures, among others (see Supplementary Table S1, panel b for details).

Four main types of costs were assessed: total, primary care, hospital, and care transition costs. Table 2 shows that most studies (five out of seven) included hospital costs. Among the three studies that explored total costs,^26^^,^^31^^,^^37^ Kasteridis *et al*^37^ generate total costs based not only on primary care and hospital care, but also on mental health, community care, social care, and continuing care.

**Table 2.** Cost components by study

	**Total cost**			**Primary care costs**		**Hospital costs**	**Care transition costs**
	**Hazra 2018^31^**	**Charlton 2013^26^**	**Kasteridis 2014^37^**	**Brilleman 2014^32^**	**Brilleman 2013^13^**	**Howdon 2018^25^**	**Kadam 2013^34^**
**Primary care**							
Primary care episodes							
Clinic face-to-face visits							
Telephone contacts							
Out-of-hours encounters							
Investigations							
Medication							
Emergency consultations							
Home visits							
**Hospital**							
Acute inpatient			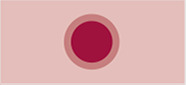				
Hospital admission	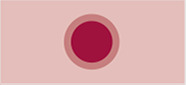	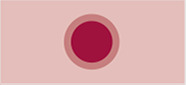				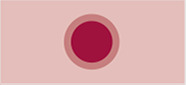	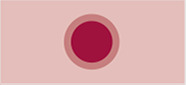
Outpatient visit	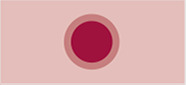	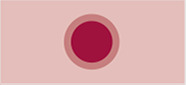				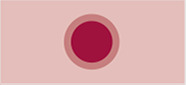	
Day case visit	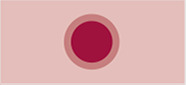	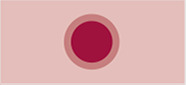	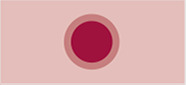			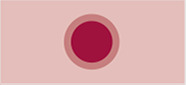	
Accident and emergency visit	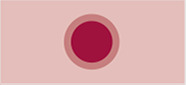	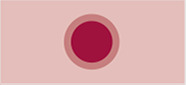	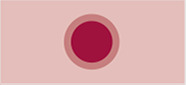			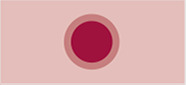	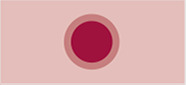
**Mental health**			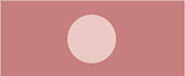				
**Community care**			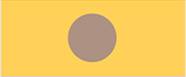				
**Social care**			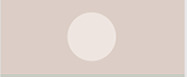				
**Continuing care**			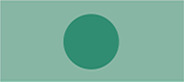				

In most studies, costs were computed by multiplying the quantity of services used by standard unit costs. The main unit cost sources included the Personal Social Services Research Unit, the General Practice Research Database, and NHS reference costs, with RESIP Gemscript Drug Dictionary and the First Databank Europe used for drug unit costs. The studies predicting hospital costs^25^ and care transition costs to hospitals^34^ used Healthcare Resource Groups.

Three studies used a longitudinal design, three used a cross-sectional design, and one used a retrospective cohort design. Four studies presented a justification for their analytic framework, including a study hypothesis (*n* = 4) or a reasoning behind the cost model specification (*n* = 1).

Regarding the statistical techniques used to model costs, three studies chose a two-part model.^26^^,^^31^^,^^37^ In the first stage, the probability of incurring positive costs is modelled. In the second stage, costs are estimated using a GLM model with a log-link and Gamma distributed errors or OLS regression with logged costs, conditional on costs being positive. Three other studies directly calibrated cost models using OLS regression (with logged or unlogged costs),^13^^,^^25^^,^^28^ and the remaining study compared OLS and a GLM model with a log-link and a Poisson distribution.^32^ Besides clinical factors (such as indicator variables for certain medical conditions), cost models typically also adjusted for age, sex, and deprivation. Only one study included a measure of functional status or age-related impairments.^31^ Four studies assessed the goodness of fit of the cost models.^25^^,^^31^^,^^32^^,^^37^

Multimorbidity is positively associated with total costs, hospital costs, and care transition costs (Table 1). Based on the results of two studies, patients with 1–3 conditions have between 1.55 and 2.85 times the mean expected total cost of individuals without any morbidity.^26^^,^^31^ The relationship between multimorbidity and primary care costs, however, does depend on the specific disease pairs that patients exhibit and their age. In other words, not all disease combinations result in higher primary care costs than treating separate patients with each condition. Depression is the main cost-increasing comorbidity across all ages, while hypertension tends to be mostly cost-limiting.^13^ Goodness of fit analyses suggest that adding multimorbidity to the specification of total or primary care cost models results in large *R*^2^ gains — *R*^2^ increased from 0.14 to 0.32 when Expanded Diagnosis Clusters (114 chronically related groupings of diagnoses) were added to an age, sex, and deprivation-only model.^32^

Only two of seven cost studies presented parameter estimates quantifying the relationship between multimorbidity and healthcare costs. Thus, a meta-analysis of cost studies was not feasible.

### Risk of bias assessment

Eight studies were considered to have the least amount of bias with valid results (good quality),^13^^,^^24^^–^^27^^,^^29^^,^^31^^,^^32^ while the remaining studies were susceptible to some biases but that were deemed insufficient to nullify their results (fair quality).^3^^,^^23^^,^^28^^,^^30^^,^^33^^–^^37^ A sample size justification was rarely provided and the exposure (in this case, multimorbidity) was only assessed once in most cases. Only five studies measured the exposure before the outcome (in this case, healthcare utilisation or costs). Loss to follow-up was only reported in one of the nine cohort studies (see Supplementary Appendix S5 for details).

### Limitations and research gaps

The main limitations discussed in the 17 studies encompass issues of data, measurement of confounders, and multimorbidity indicators. First, Hazra *et al*^31^ underscore the need to incorporate social care data into existing nationally representative datasets to create comprehensive total cost measures. Second, small-area-level social deprivation measures, which were included in most selected studies and are considered an important confounder, may cover extensive variability in socioeconomic status within a given small area and, therefore, suffer from measurement error.^24^^,^^33^ Salisbury *et al*^28^and Payne *et al*^27^ discuss the importance of accounting for disease severity. This oft-disregarded confounder can be important, as some diagnosed conditions may be inactive or have no functional status implications. Third, Brilleman and Salisbury^29^ caution against multimorbidity indicators based on QOF conditions because the primary focus is quality of care rather than chronicity. They also discuss the need to explore disease clusters of more than two conditions and to create new measures of multimorbidity calibrated on UK data.^13^^,^^32^

Other research gaps identified include exploring more detailed outcomes such as reasons for hospitalisation, regular emergency department use, or length of hospitalisation.

## DISCUSSION

### Summary

This literature review identified 17 studies that explored the healthcare costs and utilisation consequences of multimorbidity in the UK. The findings suggest that multimorbidity translates to increased healthcare costs and utilisation, including total costs, hospital costs, care transition costs, primary care use, dental care use, emergency department use, and hospitalisations. The most sizeable effect of multimorbidity is on unplanned, potentially preventable, hospitalisations, with up to 14.38 times increased odds for those with four or more conditions. This effect is independent of age.^27^ Depression is a particularly important cost and utilisation-increasing condition,^13^^,^^26^^,^^27^ and total primary care costs of multiple conditions are not purely additive, but depend on specific disease combinations and age groups.^13^

### Strengths and limitations

This study brings together the UK literature on the statistical and econometric modelling of cost and health service utilisation associated with multimorbidity. As part of BCSC, the identification of the initial set of relevant studies included a systematic literature review to minimise bias in study choice. This was supported by a clear set of inclusion and exclusion criteria throughout the search methodology, from the systematic literature review to the citation and reference review of the initial pearls. However, the authors’ choice to maximise the generalisability of findings across disease conditions meant that studies that focused on the effect of multimorbidity on a single disease patient population were excluded. A second limitation is that, even though studies from a single country were gathered, considerable heterogeneity across studies in their populations, conditions included in the multimorbidity measures, and statistical techniques was observed; the utilisation meta-analysis results should therefore be considered with caution. Finally, the applicability of the results to other countries may be limited, but their country-specific focus aims to better inform UK healthcare policy.

### Comparison with existing literature

The results of this UK-focused review concur with Lehnert *et al*’s study,^11^ which was based on 35 non-UK international studies. Multimorbidity is positively associated with healthcare costs and utilisation, with a particularly large effect on hospital stays. However, a shift in the conceptualisation of multimorbidity from purely disease counts to specific disease combinations/clusters and the focus on specific age groups are trends noted in this review. By using a less conventional search strategy, this review brings together 17 new UK-specific studies and comprehensively summarises the magnitude of the relationship between multimorbidity and healthcare utilisation and costs.

### Implications for research and practice

Conceptual frameworks describing how multimorbidity affects healthcare costs and utilisation that consider clinical, behavioural, and environmental factors, such as the one developed by Zulman and colleagues^6^ on comorbidity interrelatedness and quality of care, should more often guide statistical and econometric modelling of these outcomes. The impact of disease severity, diagnosis sequence, and quality of care on costs of patients with multimorbidity remains mostly unexplored, as well as polypharmacy and the risk of medication adverse events. Identifying the most common disease clusters has also been recognised by Whitty and colleagues^38^ as essential to advance towards a cluster-medicine model that successfully combines specialist and generalist care. Multimorbidity often worsens quality of life and disability, which are only partially captured by primary and secondary healthcare data. A comprehensive measurement of multimorbidity healthcare utilisation and costs requires social care data to be integrated into existing nationally representative datasets.

NHS England policy^39^ supports the expansion of integrated care schemes, particularly those with better coordinated community health, mental health, and hospital services. This review provides evidence in support of this policy goal by identifying depression as the main cost-increasing condition and highlighting the substantial contribution of multimorbidity to unplanned hospitalisations.
